# Clinical application of spectral CT perfusion scanning in evaluating the blood supply source of portal vein tumor thrombus in hepatocellular carcinoma

**DOI:** 10.3389/fonc.2023.1348679

**Published:** 2024-01-17

**Authors:** Chunhan Pan, Feng Dai, Liuli Sheng, Kang Li, Wei Qiao, Zheng Kang, Xiuming Zhang

**Affiliations:** ^1^ Department of Radiology, The Affiliated Cancer Hospital of Nanjing Medical University, Jiangsu Cancer Hospital and Jiangsu Institute of Cancer Research, Nanjing, China; ^2^ Department of Intervention, The Second Hospital of Nanjing, Nanjing, China; ^3^ Department of Radiology, Jiangsu Cancer Hospital, Jiangsu Institute of Cancer Research and The Affiliated Cancer Hospital of Nanjing Medical University, Nanjing, China

**Keywords:** PVTT, hepatocellular carcinoma (HCC), spectral computed tomography, spectral based image, virtual monoenergetic images

## Abstract

**Purpose:**

To evaluate the characteristic of blood supply of liver portal vein tumor thrombus (PVTT) using perfusion indexes and spectral parameters.

**Methods:**

Between July 2020 and December 2022, the study enrolled 25 liver cancer patients completed with PVTT (male=20, female=5; age 41-74 years (59.48 ± 9.12)) from the Interventional Department of Jiangsu Cancer Hospital. There were 11 cases of type III PVTT, 12 of type II PVTT, and 2 of type I PVTT (Cheng’s classification). All patients underwent spectral perfusion scans through dual-layer spectral detector computed tomography. The PVTTs were divided into proximal and distal groups based on the distance between the tumor thrombus and the main portal vein. The perfusion analysis was performed on the 120-kVp conventional images to generate hepatic perfusion index (HPI). The spectral based images (SBIs) during the artery and venous peak phases were extracted from the perfusion data. The iodine map and 40&100-keV virtual monoenergetic image (VMI) were generated from SBI data. HPI, iodine concentration (IC), CT value at 40 and 100-keV, and spectral slope (40-100keV) of the primary lesion, proximal and distal PVTT, and liver parenchyma were measured and compared. The correlation between the primary lesion and proximal and distal PVTT was analyzed.

**Results:**

The IC and spectral slope during the arterial and venous peak phases and HPI of the primary lesion, proximal PVTT, and distal PVTT were highly correlated (P<0.001). The differences between the IC and spectral slope during the arterial and venous peak phases and HPI of the primary lesion, proximal PVTT were statistically significant (P<0.001). The differences between the IC during venous peak phase and HPI of primary lesion, distal PVTT were statistically significant (P<0.001), and there was no statistically significant difference in arterial phase IC, arterial and venous phase spectral slopes.

**Conclusion:**

The IC, slope, and HPI of the distal and proximal PVTT were highly correlated with the primary lesion, indicating that PVTT was similar to the primary lesion in the liver that they were both mainly supplied by the hepatic artery. However, there was still significant heterogeneity between the proximal PVTT and the primary lesion, while the difference in the distal PVTT was relatively small.

## 1 Introduction

Portal vein tumor thrombus (PVTT) is the most common form of vascular invasion, and its incidence ranges from 44% to 62.2% at the time of HCC diagnosis in China (1). The median survival time of HCC patients complicated with PVTT is about 2.7 months if they only receive supportive treatment (2). The local treatment of PVTT includes various methods such as transarterial chemoembolization (TACE) and iodine particle implantation surgery, and the choice of treatment plan is closely related to the blood supply of the primary lesion and PVTT. Dual-layer spectral detector CT (SDCT) has high application value in liver cancer, which can evaluate the properties and hemodynamics of primary lesions, PVTT, and liver parenchyma with multiple parameters (3). Perfusion combined with spectral detector CT can comprehensively or semi-quantitatively evaluate the blood supply of PVTT. Through energy spectrum images and quantitative analysis methods such as virtual monoenergetic image (VMI), iodine map and spectral slope, various related information of PVTT and primary lesions can be analyzed. This study used SDCT to evaluate the blood supply of PVTT, aiming to provide a basis for selecting the best treatment method for PVTT patients.

## 2 Materials and methods

### 2.1 Characteristics of patient and tumors

Between July 2020 and December 2022, the study enrolled 25 liver cancer patients completed with PVTT [male=20, female=5; age 41-74 (59.48 ± 9.12)] from the Interventional Department of Jiangsu Cancer Hospital (**Table 1**). This retrospective study was approved by the institutional review board. All patients provided written informed consent before study participation according to the institutional. The inclusion criteria were as follows: 1: patients were histopathologically diagnosed with locally advanced HCC complicated with PVTT, or the patients had three diagnostic criteria: cirrhosis, typical imaging manifestations of HCC and elevated AFP; 2: patients showed adequate renal function with a serum creatinine level of no more than 2.0 mg/dL (177 umol/L); 3: patients had no history of iodine allergy. 4: patients had not received any treatment related to HCC.

**Table 1:** Clinical factors of patients.

Clinical factors	No. (%)
Sex	
Male	20 (80)
Female	5 (20)
Age (years)	
40-50	2 (8)
50-60	10 (40)
>60	13 (52)
PVTT classification	
Type I	2 (8)
Type II	12 (48)
Type III	11 (44)
Type IV	0

### 2.2 CT scanning parameter

A clinical SDCT scanner (IQon, Philips Healthcare, Best, The Netherlands) was applied in this research, and 25 patients were scanned in a head-first, supine position. A body weight-adapted volume of a non-ionic, iodinated contrast agent (iohexol 350 mg/mL) was administered intravenously via the peripheral vein at a mean flow of 3.5 mL/s, followed by a 30 mL of saline flush. The perfusion scanning range was centered around the lesion, covering a total of 8cm and included five cycles of cyclic scanning. Phantom scans were performed five times through the 100 mAs of fixed tube current. The additional scan parameters used in patients and phantom scans were collimation at 64 × 0.625 mm, rotation time at 0.5s, pitch 0.671, and tube voltage at 120 keV.

### 2.3 Region of interest (ROI) design and image reconstruction

**Figure 1:**
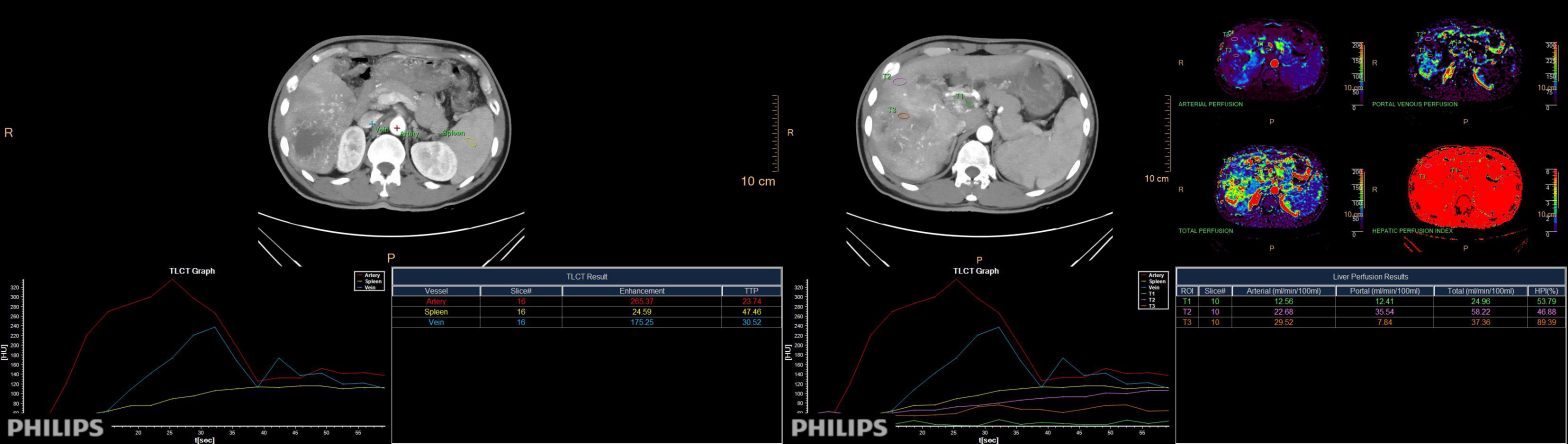
The peak time curve and perfusion analysis diagram of arteries, veins, and spleen. Directly obtain the HPI value of the region of interest through measurement.

**Figure 2:**
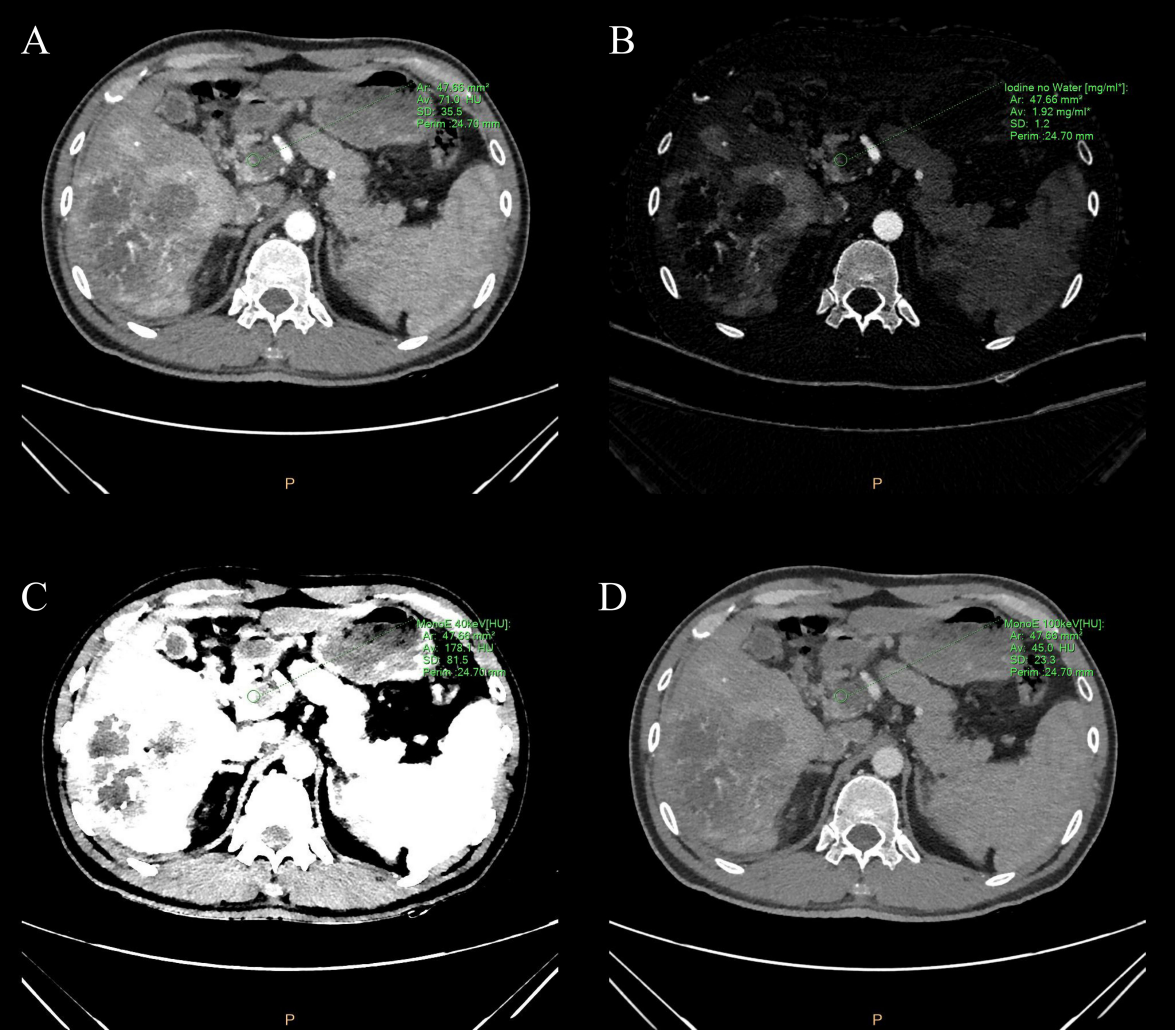
**(A)** Conventional image, **(B)** iodine map image, and **(C, D)** 40&100-keV virtual monoenergetic images of the portal vein tumor thrombus during the peak arterial phase. The green circle represents the measured ROI in the portal vein tumor thrombus. ROI is plotted by using spectral CT post-processing software. The green letters around are some spectral parameters.

During the arterial and venous peak phases of the CT scanning (**Figure 1**), the circular ROIs of at least 500 mm² were placed on the primary lesion, proximal and distal PVTT, and liver parenchyma. To minimize any measurement error from different ROIs, we took the average value of three measurements. The ROIs of liver parenchyma and primary lesion were measured at the same level. ROIs did not include nonrepresentative structures like blood vessels, bile ducts, and lymph nodes. The perfusion analysis was performed on the 120-kVp conventional images to generate perfusion results (like hepatic perfusion index (HPI) (**Figure 1**).The spectral based images (SBIs) at the artery and venous phases were extracted from perfusion data. The iodine map and 40-100 keV VMI were generated from SBI, at a reconstructed slice thickness of 1 mm and an increment of 1 mm. The iodine concentration (IC), CT value at 40 and 100-keV, and spectral slope (40-100keV) of the primary lesion, proximal and distal PVTT, and liver parenchyma were measured (**Figure 2**).

### 2.4 Statistical analysis

The SPSS version 26.0 (SPSS, Inc., Chicago, IL, USA) was used for data analysis. The measurement data was described as mean ± standard deviation, and the K-S test was used to evaluate whether the measurable variables exhibit a normal distribution. A scatter plot was created to observe whether its distribution was linear. Pearson correlation analysis was used to evaluate the correlation between each group of data for normal distribution variables. Paired samples were subjected to paired sample T-test, and the mean difference between groups was analyzed. The statistical significance was defined as *P* value of no more than 0.05.

## 3 Results

**Figure 3:**
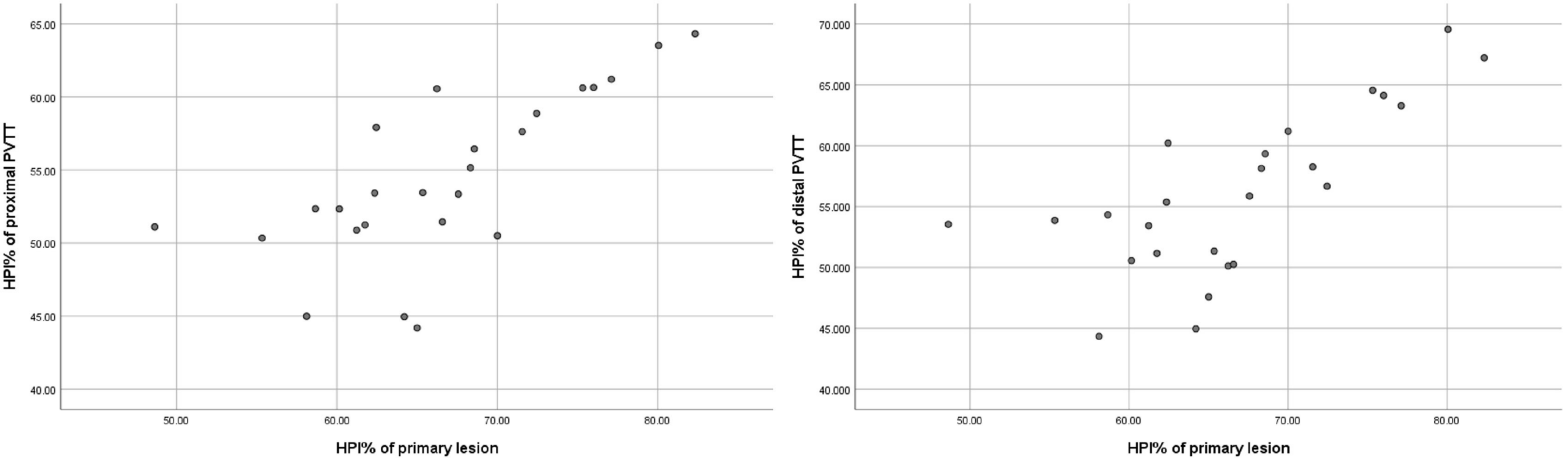
The scatter plot of HPI% from the primary lesion and proximal and distal PVTT.

**Figure 4:**
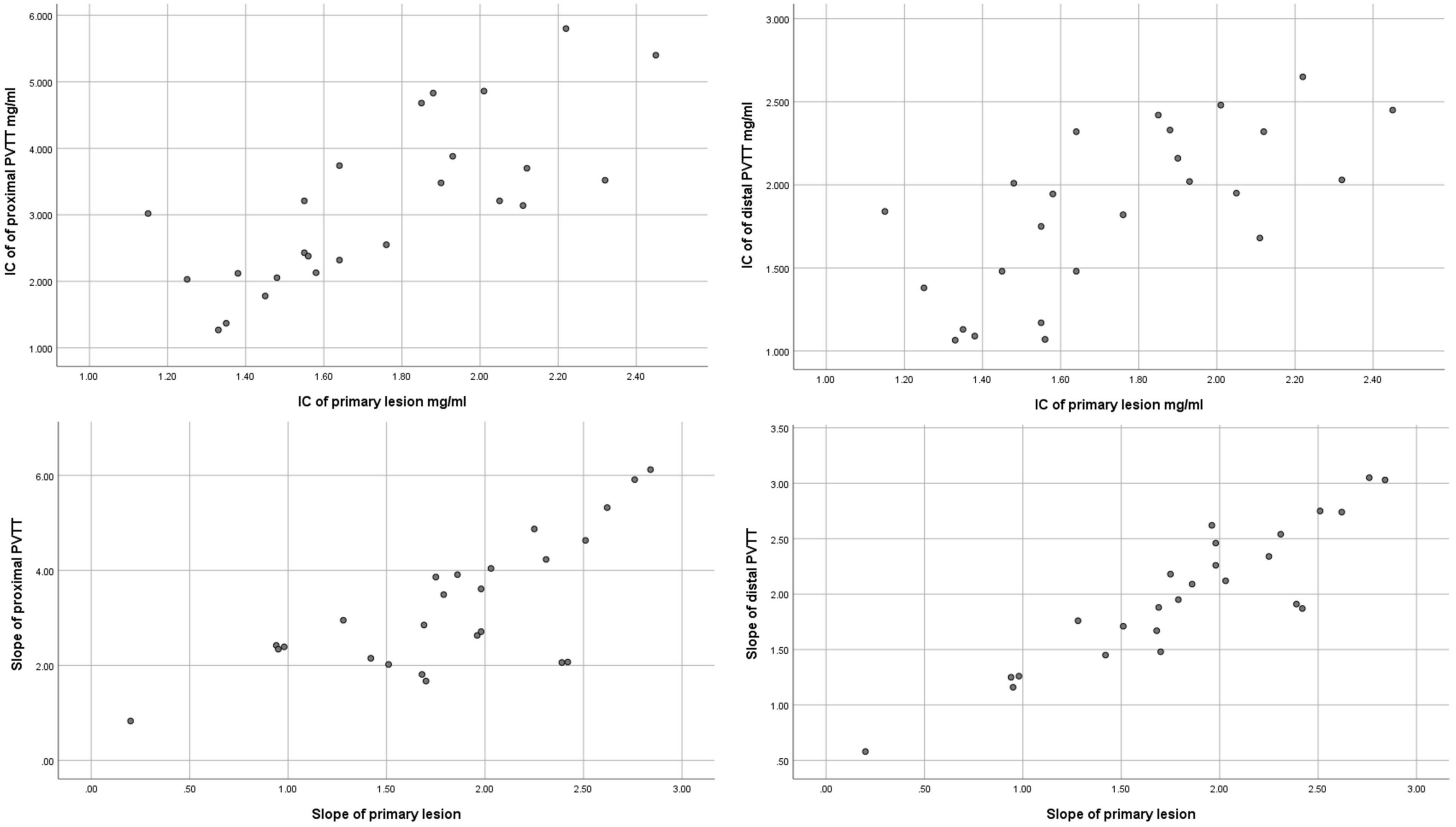
The scatter plot of IC from the primary lesion and proximal and distal PVTT during the peak arterial phase. The scatter plot of slope from the primary lesion and proximal and distal PVTT during the peak arterial phase.

The IC and spectral slope during the arterial and venous peak phases and HPI of the primary lesion, proximal PVTT, and distal PVTT were highly correlated (P<0.001) (**Table 2**; **Figures 3**, **4**). The differences between the IC and spectral slope during the arterial and venous peak phases and HPI of the primary lesion, proximal PVTT were statistically significant (P<0.001) (**Table 3**). The differences between the IC during venous peak phase and HPI of primary lesion, distal PVTT were statistically significant (P<0.001), and there was no statistically significant difference in arterial phase IC, arterial and venous phase spectral slopes (**Table 4**).

**Table 2:** Correlation analysis of primary lesion, proximal and distal PVTT.

	Primary lesion	Proximal PVTT	Distal PVTT	Correlation P
IC (arterial)	1.74 ± 0.35	3.16 ± 1.24	1.84 ± 0.50	<0.001
IC (venous)	1.88 ± 0.42	2.07 ± 0.30	1.73 ± 0.32	<0.001
Slope (arterial)	1.83 ± 0.64	3.24 ± 1.38	2.00 ± 0.62	<0.001
Slope (venous)	2.16 ± 0.59	2.42 ± 0.41	2.13 ± 0.51	<0.001
HPI (%)	66.61 ± 7.92	54.46 ± 5.62	55.97 ± 6.64	<0.001

**Table 3:** Paired t-test between primary lesion and proximal PVTT.

	Primary lesion	Proximal PVTT	95%CI	t	P
IC (arterial)	1.74 ± 0.35	3.16 ± 1.24	-1.42(-1.83, -1.00)	-7.08	<0.001
IC (venous)	1.88 ± 0.42	2.07 ± 0.30	-0.18(-0.25, -0.11)	-5.42	<0.001
Slope (arterial)	1.83 ± 0.64	3.24 ± 1.38	-1.40(-1.82, -0.99)	-6.92	<0.001
Slope (venous)	2.16 ± 0.59	2.42 ± 0.41	-0.26(-0.38, -0.14)	-4.57	<0.001
HPI(%)	66.61 ± 7.92	54.46 ± 5.62	12.15(9.89, 14.41)	11.11	<0.001

**Table 4:** Paired t-test between primary lesion and distal PVTT.

	Primary lesion	Distal PVTT	95%CI	t	P
IC (arterial)	1.74 ± 0.35	1.84 ± 0.50	-0.10(-0.25, 0.05)	-1.41	0.172
IC (venous)	1.88 ± 0.42	1.73 ± 0.32	0.16(0.08, 0.24)	4.19	<0.001
Slope (arterial)	1.83 ± 0.64	2.00 ± 0.62	-0.17(-0.28, -0.06)	-3.17	0.004
Slope (venous)	2.16 ± 0.59	2.13 ± 0.51	0.03(-0.08, 0.14)	-0.59	0.559
HPI (%)	66.61 ± 7.92	55.97 ± 6.64	10.65(8.36, 12.93)	9.63	<0.001

## 4 Discussion

### 4.1 The treatment methods of PVTT

The primary causes of PVTT development include gene mutation, active replication of HBV, and direct invasion of the portal vein by tumor (4–6). The Barcelona Clinic Liver Cancer guidelines classify HCC accompanied by PVTT as advanced stage (BCLC C stage), and such patients are only eligible for palliative systemic treatment (7) Despite the varying burden of HCC faced by various countries, more proactive management methods are proposed for advanced HCC cases (8–10), including local, locoregional and systemic therapies. Local therapies include surgery (liver resection and transplantation) and radiotherapy the 3-dimensional conformal radiotherapy (3D-CRT), external beam radiotherapy (EBRT)) (10).Locoregional therapies include transarterial chemoembolization (TACE), transarterial radioembolization (TARE), and hepatic artery infusion chemotherapy (HAIC) (11). Systemic therapies include immunotherapy with nivolumab, pembrolizumab and others, and targeted therapy with sorafenib, regorafenib, Lenvatinib etc (12). The use of drugs combined with other methods like radiation therapy (RT), TACE, TARE, and HAIC can effectively control HCC development. However, the main treatment method has always been a focus of clinical discussion, and various treatment methods through hepatic artery perfusion need to clarify whether the target lesion has hepatic artery blood supply and the degree of hepatic artery blood supply, which will directly affect the efficacy of TACE for lesion treatment and whether other methods need to be combined.

### 4.2 The application of CT in PVTT evaluation

Perfusion CT can objectively and quantitatively evaluate the blood perfusion of the tumor and its adjacent tissues (13). SDCT can offer energy spectrum images and quantitative analysis methods in liver diseases, such as virtual mono energy images (VMIs), virtual plain scans, iodine density maps, atomic number maps, and energy spectrum curves; it can further provide valuable information for disease localization and qualitative diagnosis (14). Moreover, it can provide more tissue characteristics beyond a conventional CT scanner (15). The function of spectral slop (40-100 keV) is similar to spectral attenuation curves, it can plot the CT numbers of tissues at different energy levels of VMIs. Although hepatic arteries are not the main blood supply to the liver, they are the dominant blood supply to liver tumors due to neoplastic angiogenesis (16), which is the basis for the TACE. Our research showed that the blood supply of PVTT was similar to that of liver cancer lesions, and both were primarily supplied by arteries, which was of great significance for selecting the optimal treatment method for advanced HCC patients complicated with PVTT. Moreover, this study found that the mean HPI value was significantly higher in the primary lesion than in the liver parenchyma, and the high HPI values could explain the reduction of the portal perfusion inflow and a simultaneous increase in arterial inflow in the primary lesion, which further validated the reliability of the selection of control criteria in this study. The difference in mean values between the primary lesion and proximal PVTT was statistically significant, while there was a statistically significant difference in IC values and HPI between the primary lesion and distal PVTT during the venous peak period. There was no statistically significant difference in mean values between the remaining items. PVTT might obstruct the portal vein blood flow of the tumor (17) and the corresponding segment of liver parenchyma to influence perfusion parameters. Nevertheless, the blood supply of the distal PVTT was less affected by the main portal vein, so the values of IC and spectral slope from the distal PVTT were similar.

### 4.3 Selection of Treatment plans guided by PVTT blood supply evaluation

According to the guidelines for HCC in China, patients with liver cancer combined with PVTT can apply TACE, systemic therapy, radiotherapy, chemotherapy, or surgical resection according to the situation. Surgical resection is widely recognized as a curative treatment measure, but it can only be targeted at patients with PVTT I-II who have good liver function. Various guidelines agreed that TACE was the preferred and effective palliative treatment for advanced liver cancer (18, 19). Chung et al (20) enrolled 83(66.4%) of the 125 advanced HCC patients complicated with PVTT who were treated with TACE and 42 (33.6%) who received supportive care. They found that repeated TACE had more significant survival benefits than supportive care in treating patients with Child-Pugh class A HCC. With the diversification of treatment methods, Li et al. (21) conducted a meta-analysis that included seven studies with 1,018 patients, in which 602 patients received TACE and I^125^ irradiation stent placement and 416 underwent TACE and stent placement without endovascular brachytherapy (EVBT).The study found that I^125^ irradiation stent could improve the cumulative stent patency rate in 6 and 12months and the survival rate in 12 and 24months, indicating that the I^125^ radiation stent was more effective in treating HCC with PVTT and TACE combined with EVBT may become an alternative treatment method for HCC with PVTT. Kim et al. found that HCC patients complicated with PVTT who received TACE with RT had a longer median time to progression and OS than those who got TACE or sorafenib (*P* < 0.001) (22).

Based on the above studies, it was found that HCC combined with PVTT still needed to be combined with other different treatment methods on the basis of TACE, which was basically consistent with the results of this study. This study showed that PVTT was similar to the primary lesion which was mainly supplied by the hepatic artery, and TACE was effective. However, the efficacy of TACE in treating PVTT was not as good as that of primary lesions. As a result, the author believed that although PVTT was mainly supplied by the hepatic artery, there was still significant heterogeneity between proximal PVTT and primary lesions. Most parameters of distal PVTT were not statistically different from primary lesions, but the difference in HPI was still significant. Therefore, TACE alone was not effective in treating PVTT. Therefore, considering the blood supply characteristics of PVTT, a comprehensive treatment plan combined with TACE should become the mainstream treatment method for patients with advanced hepatocellular carcinoma accompanied by portal vein tumor thrombus. Before selecting the treatment plan, performing spectral perfusion analysis on PVTT to evaluate its hepatic artery blood supply and the degree of difference with liver cancer and choosing an appropriate combination plan may have high reference value for improving the treatment efficacy.

In summary, both PVTT and primary lesions were supplied by the hepatic artery, but the degree of blood supply and tumor tissue still had certain heterogeneity compared to the liver cancer. A comprehensive treatment plan based on TACE combined with various methods should become the mainstream treatment method for patients with advanced hepatocellular carcinoma and portal vein cancer thrombus.

### 4.4 Limitation

This study used a clear proportion of blood supply to primary liver cancer lesions as a reference standard, and the evaluation method mainly relied on imaging, which was relatively single. In the later stage, DSA angiography results will be considered to further clarify the conclusions of this study. If part of patients have pathological results as support, the conclusion will be more clear. Due to the fact that the patients collected in this study are mainly with advanced liver cancer and the inclusion criteria are relatively strict, the sample size is relatively small, and the results have certain limitations. In the later stage, the sample size will be further expanded to make the conclusions more accurate.

## Data availability statement

The original contributions presented in the study are included in the article/supplementary material. Further inquiries can be directed to the corresponding author.

## Ethics statement

The studies involving humans were approved by Ethics Committee of Jiangsu Cancer Hospital. The studies were conducted in accordance with the local legislation and institutional requirements. Written informed consent for participation was not required from the participants or the participants’ legal guardians/next of kin in accordance with the national legislation and institutional requirements. Written informed consent was obtained from the individual(s) for the publication of any potentially identifiable images or data included in this article.

## Author contributions

XZ: Methodology, Project administration, Writing – original draft, Writing – review & editing. CP: Data curation, Methodology, Writing – original draft. FD: Formal Analysis, Investigation, Writing – review & editing. LS: Investigation, Writing – review & editing. KL: Investigation, Software, Writing – review & editing. WQ: Conceptualization, Methodology, Writing – review & editing. ZK: Data curation, Methodology, Project administration, Writing – review & editing.
